# Death from Chloral

**Published:** 1871-06

**Authors:** W. H. Lathrop


### *Death from Chloral.* By W. H. Lathrop, M. D.

A physician from the interior of Michigan called upon me recently, who related a case of death from chloral, that occurred in his own practice. One hundred (100) grains were given in the course of three hours.

The patient was a merchant, of the best general health, about thirty years of age, who was suffering from an attack of *delirium tremens.* He had not slept, apparently, for about six days, but was not in any respect delirious at the time of giving the medicine, nor had he been so. The patient was perfectly sensible in his conversation, and urged an increase in the quantity of his medicine, in order that he might obtain a little sleep. The first dose of twenty grains was given at noon; successive doses of the same quantity were given at 12:30 and at 1 p. m. The patient having then taken sixty grains without any apparent effect, a consulting physician was called, who was decidedly of the opinion that more chloral had better be given. Accordingly, at 2:30 p. m., another dose of twenty grains was administered without effect, followed at 3 p. m. by twenty grains more. About ten minutes after this the two physicians left the house, having noticed no change in the condition of their patient, except that he complained of a slight paralysis in the right lower extremity. They had hardly left the house, however, when they were called back to find the patient *dead.*

A *post-mortem* examination revealed nothing, except that the heart was slightly shriveled. The brain and spinal chord were carefully examined, as well as the thoracic and abdominal organs.

It does not appear from the account given of this case that any other explanation can be given of the cause of death than is afforded by the administration of the chloral.

Dr. Beech, of Coldwater, Michigan, recently reported in the *Medical Advance* the administration of 150 grains of chloral to a young lady seventeen years of age, in which recovery followed. In this case 120 grains were given at a single dose. A case in which recovery followed the administration of 270 grains in two hours was recently reported, I think, in the *Medical and Surgical Reporter.*

We are driven to the conclusion, therefore, with regard to chloral, that a given dose cannot be relied upon to produce a given effect. It is certainly a very dangerous medicine, and should be given with great caution. It would seem to resemble chloroform in its uncertainty of action, since, in administering the latter medicine, surgeons, after long experience, are often surprised by the sudden death of a patient.

